# Profile of transversal skills of Nursing students to intervene in disaster situations

**DOI:** 10.1590/0034-7167-2021-0760

**Published:** 2022-07-18

**Authors:** Paulo Alexandre Figueiredo dos Santos, Isabel Cristina Mascarenhas Rabiais, Leila Miriam Conde Faria Sales, Carolina Miguel Graça Henriques

**Affiliations:** I Escola Superior de Saúde da Cruz Vermelha Portuguesa. Lisboa, Portugal; II Universidade Católica Portuguesa. Lisboa, Portugal; III Instituto Politécnico de Leiria, Escola Superior de Saúde de Leiria. Leiria, Portugal

**Keywords:** Students, Nursing, Disaster Planning, Professional Skill, Nursing, Education, Surge Capacity., Estudiantes de Enfermería, Gestión de Desastres, Competencia Profesional, Educación en Enfermería, Capacidad de Respuesta ante Emergencias., Estudantes de Enfermagem, Administração de Desastres, Competência Profissional, Educação em Enfermagem, Capacidade de Resposta ante Emergências.

## Abstract

**Objective:**

to identify the profile of transversal teaching/learning skills, which allows an adequate intervention of disaster nursing students.

**Methods:**

this is research framed in the qualitative, transversal paradigm methodology, supported by inductive and exploratory reasoning.

**Results:**

it was found that there is no consensus between Nursing Education Major Coordinators/Directors and disaster nursing experts regarding transversal skills considered decisive, due to the current absence of the theme in study plans and the limited personal and professional training of professors.

**Final considerations:**

the development and implementation of teaching/learning strategies that allow the development of transversal skills, favoring students’ ability to meet this reality in a conscious, balanced and efficient way, is crucial.

## RESUMO

**Objetivo:**

identificar o perfil de competências transversais de ensino/aprendizagem, que permita uma intervenção adequada do estudante do Curso de Licenciatura em Enfermagem no domínio da catástrofe.

**Métodos:**

investigação enquadrada na metodologia do paradigma qualitativo, transversal, apoiada no raciocínio indutivo e exploratória.

**Resultados:**

verificou-se que não existe consenso entre os Coordenadores/Diretores dos Cursos de Licenciatura em Enfermagem e os peritos de enfermagem em catástrofe quanto às competências transversais consideradas determinantes, decorrente da atual ausência da temática nos planos de estudo e da diminuta formação pessoal e profissional dos docentes.

**Considerações finais:**

o desenvolvimento e implementação de estratégias de ensino/aprendizagem, que permitam o desenvolvimento de competências transversais, favorecendo a capacidade do estudante para se encontrar com esta realidade de maneira consciente, equilibrada e eficiente, assume-se crucial.

## RESUMEN

**Objetivo:**

identificar el perfil de competencias transversales de enseñanza/aprendizaje, que permita una adecuada intervención del estudiante de la Licenciatura en Enfermería en el dominio de la catástrofe.

**Resultados:**

se constató que no existe consenso entre los Coordinadores/Directores de la Licenciatura en Enfermería y los expertos en enfermería de catástrofes respecto a las competencias transversales consideradas cruciales.

**Métodos:**

investigación enmarcada en la metodología del paradigma cualitativo, transversal, sustentada en razonamientos inductivos y exploratorios.

**Consideraciones finales:**

es crucial el desarrollo e implementación de estrategias de enseñanza/aprendizaje que permitan el desarrollo de competencias transversales, favoreciendo la capacidad del estudiante para afrontar esta realidad de manera consciente, equilibrada y eficiente.

## INTRODUCTION

From a health-centered perspective, a disaster is a situation of sudden, unexpected and excessive demand for emergency health care that exhausts all available resources^(1)^. Several authors assume that, in a disaster situation, responses have to be organized in order to translate the awareness reached by the various professionals and authorities in general on the specific complexity of the event and on the need to address it in as many aspects as possible in the fields of human knowledge^(2)^.

It is initial training, which aims to promote solid technical-scientific, humanistic and ethical training, in order to fit the skills profile defined by the Order of Nurses, which could form the basis for the development of a set of common skills for nurses, in this specific field, as well as the definition and recognition of specific added skills of the different fields of specialization in nursing, in the disaster field^(3)^. However, intervention in disaster situations, complex and singular events, susceptible to triggering in health professionals’ internal conflicts and uncertainties, leads to the question of whether nursing students in the first cycle have sufficient resources - cognitive, interpersonal and systemic - to respond efficiently to such a level of demand. The cognitive structure of students has the ability to consolidate new knowledge; however, it is necessary to have pre-existing bases acquired during the teaching-learning process that serve as a substrate for the transfer of this knowledge to practice^(4)^.

Hence, specifically in the disaster field, Kalanlar^(5)^ argues that the development of transversal skills, which allow students to develop critical thinking, promoting empowerment and autonomy, favoring the development of their creativity and fostering critical thinking is crucial. It appears that it is crucial that the learning process’ essential purpose is demonstrated in the development of students’ transversal skills, so that they can reach a maturity that makes them capable of meeting this reality in a conscious, balanced and efficient way.

## OBJECTIVE

To identify, through the perceptions of Technical-Scientific Council Chairmen or Nursing Education Major Coordinators/Directors and disaster nursing experts, what are the transversal skills, defined by the Tuning Educational Structures in Europe project - Phase I^(6)^, that allow nursing education major (NEM) students, consciously, to articulate the various dimensions inherent to the disaster field, enabling a competent performance in the exercise of their functions.

## METHODS

### Ethical aspects

The present study was approved by the Ethics Committee of the *Universidade Católica Portuguesa* - Health Sciences Institute, on March 27, 2017.

### Study design

Given the complexity of the phenomenon under study, an investigation was chosen within the qualitative paradigm methodology framework, using the triangulation of methods, as well as operational criteria, defined by the Standards for Reporting Qualitative Research (SRQR), to specifically support the data collection phase.

### Methodological procedures

Based on the underlying assumptions of the core question of research and given the complexity of the phenomenon under study, we opted for an investigation framed in the qualitative paradigm, resorting to the triangulation of methods to specifically support the data collection phase, which made it possible to transform implicit forms of knowledge into explicit forms, capable of understanding and knowing the truth in all its fullness, using discursive or analytical knowledge^(7)^. This is a cross-sectional study, mobilizing inductive reasoning and a rigorous description of phenomena based on exploratory research.

Initially, aiming at understanding the schools’ contribution in terms of technical-scientific training in promoting the development of professional skills, in disaster nursing students, we tried to identify if the theme, as a curricular unit, was integrated in the nursing courses’ curricula. For this purpose, a survey of forty study plans of the first cycle of studies in nursing was carried out, available on the official websites of nursing schools or integrated nursing courses at the university or polytechnic higher education level (Mainland, Autonomous Region of the Azores and Madeira). The results showed that, of the forty study plans, 85% do not have an integrated disaster domain. Of the schools that integrated the disaster domain in their study plan (15%), 7.5% had the typology of optional curricular unit and 7.5% have integrated the disaster domain as a mandatory curricular unit, reflecting that training in this field is still fragile and has reduced visibility in the current training framework in nursing. However, since through the assignments assigned to some curricular units, it was not evident whether or not the disaster field was covered. These data were later confirmed in the course of semi-structured interviews with Technical-Scientific Council Chairmen or NEM Coordinators/Directors.

The interviews proved to be fundamental to clarify whether, from participants’ point of view, the study plans in operation in schools allow NEM students to develop the necessary skills to know how to act effectively and efficiently in a disaster situation. Subsequently, the same guide was applied to a group of experts in the disaster field, using the focus group technique, which made it possible to reconcile uniformity with diversity, and thus to find consensus on the study objectives^(8)^.

The interview script was structured in three thematic dimensions. The first dimension included an introductory part in which there was a brief presentation of the researcher and explanation of the study. The second dimension presented the sociodemographic variables to characterize the participants and four open questions to be explored. The choice in the use of open-ended questions, according to Silverman^(8)^ allows respondents to respond using their own vocabulary, which translates into more consistent investigations. The third, consisting of a Likert-type questionnaire, included five response categories (1 - Not at all important; 2 - Not at all important; 3 - Important; 4 - Very important and 5 - Extremely important) to measure the perception of Technical-Scientific Council Chairmen or NEM Coordinators/Directors and disaster nursing experts, from the core skills defined by the Tuning Educational Structures in Europe project - Phase I^(6)^ about which skills they considered essential for students to intentionally mobilize, in order to know how to act effectively and efficiently in a disaster situation. One of the advantages of this instrument is the uniformity of its presentation. The questions were always presented in the same sequence, with the same instructions, ensuring uniformity of measurement conditions, guaranteeing fidelity and facilitating comparison between subjects^(8)^.

### Sample

Sampling was intentional, divided into two groups. Firstly, participants’ sociodemographic and professional characteristics was carried out, in order to obtain a better understanding of the characteristics that may be relevant to data analysis and interpretation, such as sex, age, educational qualifications, job tenure as NEM Coordinator/Director, field of training of the third cycle of studies, number of missions carried out and field of specialization. In the selection criteria of participants, the importance of personal characteristics considered necessary to obtain rich descriptions on the subject under analysis was safeguarded, such as the ability to express themselves clearly, provide information and reflect on the topic. Technical-Scientific Council Chairmen or Coordinators/Directors of forty NEM were selected, since professors are responsible for implementing the learning objectives, taking into account the curricular objectives, continuity and management of contents to be taught. However, only thirty-five schools participated in the study, since two schools did not express interest in participating, and, from three others, it was not possible to obtain a response to the invitation addressed in a timely manner.

In order to analyze whether the perceptions of Technical-Scientific Council Chairmen or NEM Coordinators/Directors were convergent or divergent from the perceptions of disaster nursing experts, the focus group method was used. A group of six participants was selected, in which the selection criteria were based on their knowledge and experience in intervening in disaster scenarios, allowing the sharing and comparison of their experiences in the production of new knowledge, as the experience and being in the context allow a reflection and a better internalization of all skills that nurses must acquire and develop. In carrying out the focus group, an online communication software, Skype^®^, version five, was used, as some participants were on a humanitarian mission.

### Data collection and analysis

From the data obtained through semi-structured interviews and the focus group using the recording technique, content analysis was carried out, considered a research technique that allows valid inferences to be made. Data analysis and organization validation was structured in categories and identification of registration units, considering certain principles, such as completeness and exclusivity, representativeness, homogeneity, and productivity^(9)^. With regard to transferability, this criterion was ensured by using a sample of participants who are related to the phenomenon under study, with a view to increasing the possibility of finding precise information on it. In the dependence criterion, an attempt was made to audit the process and the investigation method itself, transferring to the methodological dimension the entire research process’ detailed documentation, as well as methodological decisions, so that other researchers are able to follow the investigation process. In confirmability, it was always intended that the relevant data from the study would be a product of the investigation and a clear attempt not to bias the researcher himself.

## RESULTS

From the data collected and the text skimming of the speeches of Technical-Scientific Council Chairmen or NEM Coordinators/Directors and disaster nursing experts, it was possible to identify common elements that were grouped into two axes: *Teaching-learning processes in the development of skills in the disaster field; Intervention of general care nurses in disaster situations*.

In the first axis of analysis, it was intended, through the different categories, to find revealing references, in order to outline the dominant tendencies in the discourses regarding the different participants’ positioning in relation to the importance and necessity of including the disaster domain in the course curricula. Regarding the second axis of analysis, it focuses on: the importance of a more objective regulation of students’ skills; the profile of skills considered essential for the development of competent professional and human performance of general care nurses in disaster situations; what have been the conditions for including the theme in the educational plan, as well as whether students have the cognitive maturity necessary for acquiring skills in the disaster field.

These axes of analysis that emerged gave rise to the system of eight categories that were used to interpret the participants’ discourse, which are positioned in the different axes as follows (Chart 1).

**Chart 1:** Axes and categories

**Theme**	**Category**
Teaching-learning processes in the development of skills in the disaster field	Diagnose in order to train: the training needs of NEM students in the disaster field
	Academy: implications for the teaching/learning process of NEM students in the disaster field
	Inter-institutional and transdisciplinary cooperation: the importance in the pedagogical action of NEM students in the disaster field
	Use of advanced simulated practice in disaster field teaching/learning
Intervention of general care nurses in disaster situations	Regulation of formative content in nursing (subject) disaster (field)
	General care nurse skills profile for a systematized response in a disaster situation
	Reconfiguration of professional culture
	Cognitive maturity of NEM students for a systematized response in the disaster domain

NEM: nursing education major.

However, of the eight emerging categories, only the structure of a category related to the objective initially defined is presented, i.e., to understand the current functional content of transversal skills, defined by the Tuning Educational Structures in Europe - Phase I^(6)^ which are considered relevant to be included in the study plans. Regarding the results of the application of a psychometric response scale (Likert scale) to NEM Coordinators/Directors and their respective disaster experts, there was a lack of consensus regarding the profile of skills to be defined (Table 1).

**Table 1:** Mean of the Likert-type scale questions’ scores of nursing education major coordinators/directors and disaster nursing experts

**Question/skill**			**M**	**σ**	**Mo**	**Md**	**Xmín.**	**Xmax.**
Q1	Individual capacities for thinking in action	NEM coord/dir	4.57	.65	5.00	5.00	3.00	5.00
		Expert nurses	3.83	1.47	5.00	5.00	2.00	3.00
Q2	Ability to have confidence and clarity in autonomous decision-making	NEM coord/dir	4.74	.44	5.00	5.00	4.00	5.00
		Expert nurses	2.83	.41	3.00	3.00	2.00	3.00
Q3	Ability to have an impartial and reality-adjusted representation of oneself in order to anticipate how one is able to behave or react in this or that situation	NEM coord/dir	4.51	.66	5.00	5.00	3.00	5.00
		Expert nurses	3.00	.00	3.00	3.00	3.00	3.00
Q4	Ability to organize and plan time	NEM coord/dir	4.60	.60	5.00	5.00	3.00	5.00
		Expert nurses	3.00	.00	3.00	3.00	3.00	3.00
Q5	Ability to implement learning strategies	NEM coord/dir	4.26	.92	5.00	4.00	1.00	5.00
		Expert nurses	3.33	.52	3.00	3.00	3.00	4.00
Q6	Ability to promote a safe environment	NEM coord/dir	4.57	.78	5.00	5.00	2.00	5.00
		Expert nurses	5.00	.00	5.00	5.00	5.00	5.00
Q7	Ability to make decisions and solve problems	NEM coord/dir	4.34	.80	5.00	4.00	2.00	5.00
		Expert nurses	5.00	.00	5.00	5.00	5.00	5.00
Q8	Ability to use technological systems and manage information	NEM coord/dir	4.80	.41	5.00	5.00	4.00	5.00
		Expert nurses	5.00	.00	5.00	5.00	5.00	5.00
Q9	Ability to communicate	NEM coord/dir	4.26	1.01	5.00	5.00	1.00	5.00
		Expert nurses	5.00	.00	5.00	5.00	5.00	5.00
Q10	Knowledge of a second language	NEM coord/dir	4.71	.52	5.00	5.00	3.00	5.00
		Expert nurses	3.50	.55	3.00	3.50	3.00	4.00
Q11	Ability to work in groups	NEM coord/dir	4.54	.70	5.00	5.00	2.00	5.00
		Expert nurses	5.00	.00	5.00	5.00	5.00	5.00
Q12	Ability for criticism and self-criticism	NEM coord/dir	4.57	.65	5.00	5.00	3.00	5.00
		Expert nurses	3.17	.41	3.00	3.00	3.00	4.00
Q13	Ability to incorporate interdisciplinary groups	NEM coord/dir	4.57	.61	5.00	5.00	3.00	5.00
		Expert nurses	5.00	.00	5.00	5.00	5.00	5.00
Q14	Ability to communicate with experts in other fields	NEM coord/dir	4.54	1.01	5.00	5.00	1.00	5.00
		Expert nurses	2.50	1.05	2.00	2.50	1.00	4.00
Q15	Ability to appreciate diversity and multiculturalism	NEM coord/dir	4.31	.80	5.00	4.00	2.00	5.00
		Expert nurses	5.00	.00	5.00	5.00	5.00	5.00
Q16	Ability to work in an international context	NEM coord/dir	4.60	.50	5.00	5.00	4.00	5.00
		Expert nurses	4.50	.55	4.00	4.50	4.00	5.00
Q17	Ability to establish an ethical and legal commitment of the profession in the face of adverse and complex situations	NEM coord/dir	4.54	.70	5.00	5.00	3.00	5.00
		Expert nurses	5.00	.00	5.00	5.00	5.00	5.00
Q18	Ability to interconnect understanding, sensitivity and knowledge, which allow the individual to see how the parts of a whole relate and group	NEM coord/dir	4.51	.92	5.00	5.00	1.00	5.00
		Expert nurses	2.50	.55	2.00	2.50	2.00	3.00
Q19	Ability to learn	NEM coord/dir	4.69	.58	5.00	5.00	3.00	5.00
		Expert nurses	3.33	.52	3.00	3.00	3.00	4.00
Q20	Ability to adapt to new situations	NEM coord/dir	4.51	.74	5.00	5.00	3.00	5.00
		Expert nurses	5.00	.00	5.00	5.00	5.00	5.00
Q21	Ability to conceive original ideas	NEM coord/dir	4.20	.83	5.00	4.00	2.00	5.00
		Expert nurses	3.67	.52	4.00	4.00	3.00	4.00
Q22	Leadership skills	NEM coord/dir	4.37	.88	5.00	5.00	2.00	5.00
		Expert nurses	5.00	.00	5.00	5.00	5.00	5.00
Q23	Ability to understand cultures and traditions of other countries	NEM coord/dir	4.40	.65	5.00	4.00	3.00	5.00
		Expert nurses	5.00	.00	5.00	5.00	5.00	5.00
Q24	Ability for autonomous work	NEM coord/dir	4.26	.78	4.00	4.00	2.00	5.00
		Expert nurses	3.50	1.05	3.00	3.50	2.00	5.00
Q25	Ability to manage and design projects	NEM coord/dir	4.34	.97	5.00	5.00	1.00	5.00
		Expert nurses	2.83	.41	3.00	3.00	2.00	3.00
Q26	Initiative spirit	NEM coord/dir	4.29	1.07	5.00	5.00	1.00	5.00
		Expert nurses	2.83	.41	3.00	3.00	2.00	3.00
Q27	Concern for quality	NEM coord/dir	4.29	1.07	5.00	5.00	1.00	5.00
		Expert nurses	3.33	.52	3.00	3.00	3.00	4.00
Q28	Willingness to win and succeed	NEM coord/dir	4.00	1.06	4.00	4.00	1.00	5.00
		Expert nurses	2.67	.52	3.00	3.00	2.00	3.00
Q29	Investigation skills	NEM coord/dir	3.60	1.26	4.00	4.00	1.00	5.00
		Expert nurses	2.50	.55	2.00	2.50	2.00	3.00

Note: NEM: nursing education major.

**Figure 1:**
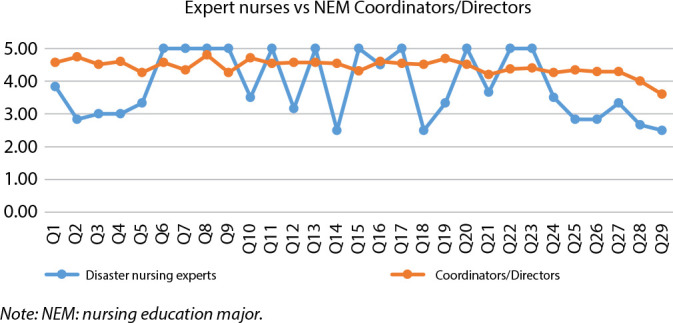
List of skills to be developed by nursing education major students in the disaster field between expert nurses and nursing education major Coordinators/Directors

In order to facilitate the reading and interpretation of the data presented in Table 1, we present Figure 1 below.

## DISCUSSION

Padilla, Creem-Regehr, Hegarty and Stefanucci^(10)^ allude that cognitive processes are responsible for thinking and, in the context of learning, lead to results in terms of knowledge, understanding and acquisition of skills, also contributing to the acquisition of information, its integration with previous knowledge, as well as the recovery of available information. These capabilities, when developed, are considered crucial for students to be able to face the complexity of modern scientific and technological life. However, according to participants’ discourses, currently students do not have these cognitive processes sufficiently developed considering the demands, constraints and challenges that disaster situations require.

[…] *in my opinion, because it is such a specific and complex field,* [...] *the first cycle student does not have the necessary maturity and experience to understand the complexity of these situations.* (S4)

[…] *the first cycle student does not have the ability to deal with uncertainty, inconsistency, contradiction, paradoxes and the imperfection of knowledge.* (S18)

[…] *the undergraduate student does not have the cognitive capacity, sufficiently developed to be able to intervene in a disaster situation* […]. (S21)

[…] *I have doubts that a first cycle student will be able to develop this path of personal and reflective appropriation of theoretical and practical knowledge, necessary to intervene in these highly complex scenarios.* (S27)

*I question whether students* [from the first cycle of studies] *have the necessary cognitive and reasoning skills that can lead them to decisions and actions to intervene in contexts of this nature* […] *I don’t think so.* (S33)

It is important that students can be prepared to use these cognitive processes in the collection, assessment and use of information for effective problem solving and sustained decision-making at personal and professional level. In the same line of thought, Tyng, Amin, Saad, Malik^(11)^ reinforce that the development of students’ transversal skills (in the form of thought or action) is the basis for the development of professional and personal maturity. It is important first of all that the domain of disaster is included in the curriculum plans, since the acquisition of knowledge positively influences learning from a cognitive-constructivist perspective, on the other hand, it is crucial that students’ training horizon in this domain is not restricted only to the technical process, which means assuming that, for students to be able to use their own internal resources and the context to guide their cognitive processes, it is necessary to develop transversal skills.

It was verified that there is no consensus between NEM Coordinators/Directors and disaster nursing experts regarding the skills considered determinant. From the analysis of Figure 1, it can be seen that NEM Coordinators/Directors consider all skills listed as relevant, with a low variability between the remaining questions’ mean scores. This fact may be associated with the perception that professors have about the fundamental premises of nursing education, in which the development of this set of skills is fundamental, in order to allow the acquisition and development of scientific and technical knowledge, skills, values and attitudes for a professional knowledge necessary for the performance of nurses’ functions and the construction of professional identity. As Mestrinho points out,

[…] the professional model that they transmit to students reveals itself as a complex skill because it involves a variety of unpredictable variables that intervene in the processes of caring for people with varying degrees of dependence in different situations^(12)^.

Disaster nursing experts, due to their ability to assess specific needs in nursing care in disaster situations and experience in these contexts, among the most valued skills, reported:

Ability to promote a safe environment - due to the unpredictability of human nature and the various pressures to which nurses are subject, the complexity and uncertainty of a subjects such as health, where the definition of error, is a reality. Specifically in disaster situations, the probability of occurrence may be exponentiated^(13)^. Hence, the development of skills that allow students to prevent, identify and control risks, adapt to different contexts and functions in scenarios of great uncertainty, with a view to the protection and safety of victims in their care, with a view to minimizing vulnerability, as well as increasing the resilience of less self-sufficient groups, becomes crucial.

- Ability to make decisions and solve problems - making the most appropriate decision in the context of disaster situations is no easy task. Lack of data and limited time are factors that interfere with clinical decision-making in these contexts. Peixoto and Peixoto^(14)^ emphasize that encouraging critical thinking through learning, in a context protected by simulated practice, based on moments of debriefing of events that occurred in the past, is a methodology that can promote critical thinking and, in this way, help students to be critically reflective, providing the opportunity to analyze problems, phenomena and concrete clinical situations in scenarios of this nature.
- Ability to use technological systems and manage information - Information technologies are currently an integral part of health professionals’ daily lives. In addition to promoting better administrative systems, simplifying communication between health professionals and interconnection with other institutions, it promotes organization and teaching, helping continuous learning and supporting nursing research. Correlating technological and information systems with the phases of disaster management, it is verified that the researches that make it possible to assess the interrelationships that can directly interfere with public health, in the sense of developing and implementing biosafety measures and integrated management of the environment and health promotion in the prevention/mitigation phase, is crucial. To the same extent that in the response phase, it serves as decision support, among the various entities involved in protection and relief operations, in the post-disaster phase, they can be used in the flow of communications, which can improve the event management^(15)^. From the above, it is necessary to train students in the use of information and communication systems, allowing to enhance knowledge in understanding operational procedures, standard and guidelines in the dissemination of information, used in this domain.
- Ability to communicate - in the health care provision, due to the constant need to exchange information, communication is, as stated by Arnold and Underman^(16)^, a vital skill. Specifically in the disaster field, by nature complex environments, communication maintenance is an essential prerequisite and the main challenge of any entity that integrates sanitary operations^(17)^. It is necessary from the framing of a risk situation to the implementation and monitoring of protection and rescue measures. It is the means that ensures the exchange of information between risk health professionals and communicating the risk appropriately to the outside world^(17)^.
- Ability to work in groups and to incorporate interdisciplinary groups - today, it is argued that the planning and organization of the response to disaster situations, awareness and human knowledge achieved by the various subjects, about the specific complexity of the event, is fundamental. The intersection between contents of the different disciplinary fields (interdisciplinarity) is crucial in the search and incorporation of scientific knowledge in this specific field^(13)^. According to Peek and Guikema^(18)^, interdisciplinarity can encourage creativity, leading the various disciplinary fields that make up the protection and rescue team, towards more conscious and consistent decision-making, greater sharing of their own knowledge, through the analysis of problems from the various perspectives of the various professionals in the team. It appears that it is necessary to develop in students an educational base that allows greater appreciation of the other subjects, the ability to communicate, to interact with the different fields of knowledge in this domain.
- Ability to work in an international context - Veenema^(13)^ argues that the internal resources of countries affected by disasters are not sufficient to ensure a rapid and effective response, requiring international assistance. Hence, health professionals sent to the field must, in principle, have an adequate profile of skills that allows them the ability to respect cultural diversity, always act with professionalism, adapt to conditions and difficulties, respect for the dignity of persons and human beings and always act in a fair and impartial manner. These abilities require a greater emphasis on teaching/learning processes in the disaster field, in order to allow students new ways of looking, contextualizing knowledge and adding new perspectives for the rethinking of nursing care in international contexts.
- Ability to establish an ethical and legal commitment of the profession in the face of adverse and complex situations - from an ethical point of view, situations of disaster should never forget the fundamental ethical principles. However, in the disaster field, nurses can be faced with ethical dilemmas, which may imply confrontations between moral principles or rules and their application in practice, a process that requires learning so that students can stand up to them with autonomy, to produce the most effective course of action. For instance, Koenig, Schultz^(19)^ point out that the processes of prioritizing victims for admission or evacuation, allocating and distributing scarce resources equitably, determining acceptable levels of care, and deciding the best plan for people who will inevitably not survive are complex. Thus, it is clear the need to strengthen the focus of educational ethics, oriented to the context of disaster, with the purpose of promoting a greater understanding and knowledge of these ethical dilemmas, allowing students, future nurses, in their care assignments and decision-making, conduct guided by reflected decisions.
- Ability to adapt to new situations - the ability to adapt can be understood as any change in the internal environment or environment that jeopardizes the survival of individuals, which may alter the balance, whether physical or psychological, and which will imply the activation of dynamic processes, in order to minimize or suppress the limitations or changes^(20)^. It appears that implicit in this capacity for adaptation is resilience, which needs to be learned, developed and perfected. The same authors reinforce that resilient individuals will have a greater capacity for adaptation, anticipation, learning, self-organization to adverse situations, allowing a better adaptability. Hence, it is crucial to encourage nursing students to develop strategies that promote the ability to achieve adaptive response patterns in the face of circumstances of high tension, such as disaster situations. These abilities (ability to recognize, diagnose and adapt to change, leadership, motivation, communication, creativity, innovation, interpersonal relationships, among others), focused on students’ potential, allow in particular contexts, of rapid, deep, intricate and disruptive changes to deal more competently with adversity and not to give in to them^(20)^.
- Leadership skills - several studies have highlighted the importance of developing nurse leadership as a central element not only for the nursing team, but also for the health team. AL-Dossary^(21)^ points out that the elements considered key in the leadership skill learning plan have to focus on aspects that can help students reach the essence of their transformation, such as the ability to motivate themselves, to control the emotions that can overwhelm their faculty of thinking, in the sense of circumventing and overcoming any barriers. On the other hand, the development of leadership skills tends to maintain high skill levels and increase the remaining team members’ performance levels.
- Cultural diversity and understanding of cultures and traditions of other countries - in an increasingly multicultural society, nurses are required to understand the multiple dimensions and specificities inherent to cultural diversity in clinical practice^(22)^. In catastrophic scenarios, nurses are expected to understand the different forms and scenarios of coexistence and relationships between different cultures and ethnic groups. The importance of promoting knowledge regarding cultural diversity in learning processes was reflected, reinforcing students’ capacities for a better cultural awareness, capable of mobilizing cultural relativity in their practice in contexts of this nature.

### Study limitations

We consider the limited number of investigations that focus on the understanding of this theme as limitations, which condition the reflection and construction of a more sustained analysis framework. On the other hand, the initial difficulties in accessing participants who are experts in the disaster field as well as some schools in accepting to participate in research.

### Contributions to nursing

The inferences raised from the data analysis constituted, in our perspective, a contribution to a greater awareness of the need to integrate the disaster domain in the NEM study plans, since the acquisition of knowledge positively influences student learning from a cognitive-constructivist perspective. It is also our opinion that this is an investigation linked to action and innovation, allowing to respond to the growing demands that society places, making possible, through the results, implications for the training practice of students and future nursing professionals, translating in turn into a reference for the five fields that structure the subject: research, practice, teaching, management (dimensions that work with emotional intelligence), and consultancy.

## FINAL CONSIDERATIONS

The information collected supports the concerns that served as the basis for this study, noting that, in addition to scientific knowledge considered a *sine qua non*, it is necessary to encourage nursing students to develop transversal skills that allow them to achieve a state of maturity that make them capable of developing flexibility, creativity, autonomy, a sense of responsibility, teamwork, adaptation to change, the ability to critically reflect, make decisions, act competently and autonomously within an interdisciplinary team. This process can be achieved through teaching, since learning must address professionalization processes, allowing the acquisition of resources to know how to act in order to construct and apply appropriate responses to professional demands, regardless of the context. Hence initial training should be perceived as a starting point for this commitment. It is the integration of these theoretical frameworks into study plans that will allow students to become aware of confronting these challenging situations, allowing them to develop and improve skills in order to respond effectively in the disaster field.
